# Supplementary figures and images for: Tableware trade in the Roman East: Exploring cultural and economic transmission with agent-based modelling and approximate Bayesian computation
Source: PLoS One. 2020 Nov 25;15(11):e0240414. doi: 10.1371/journal.pone.0240414 (PMC7688115; doi:10.1371/journal.pone.0240414)

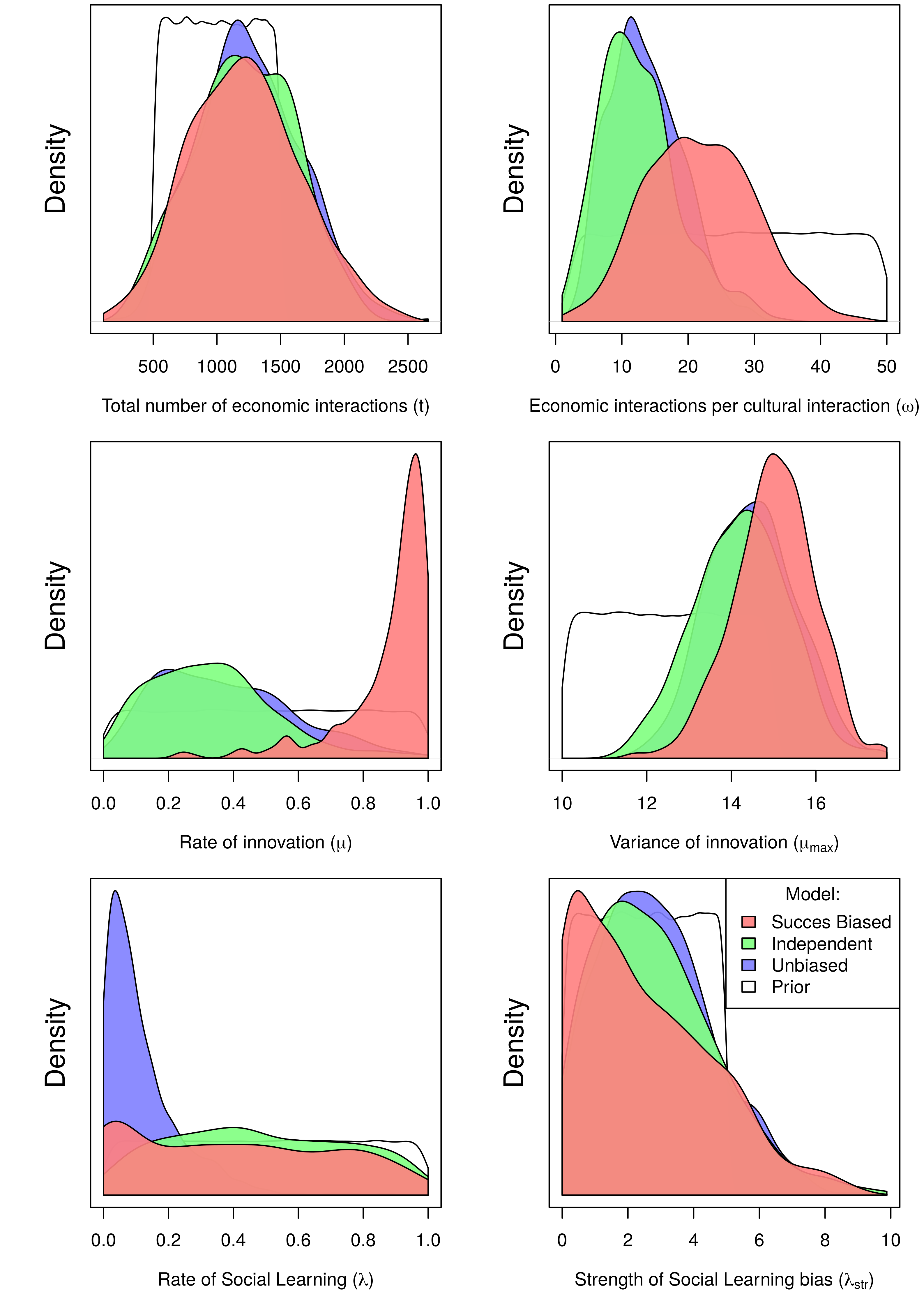

Supplement: S1 Fig — Complete posterior distributions for all parameters of the model. (TIF) [file pone.0240414.s001.tif]

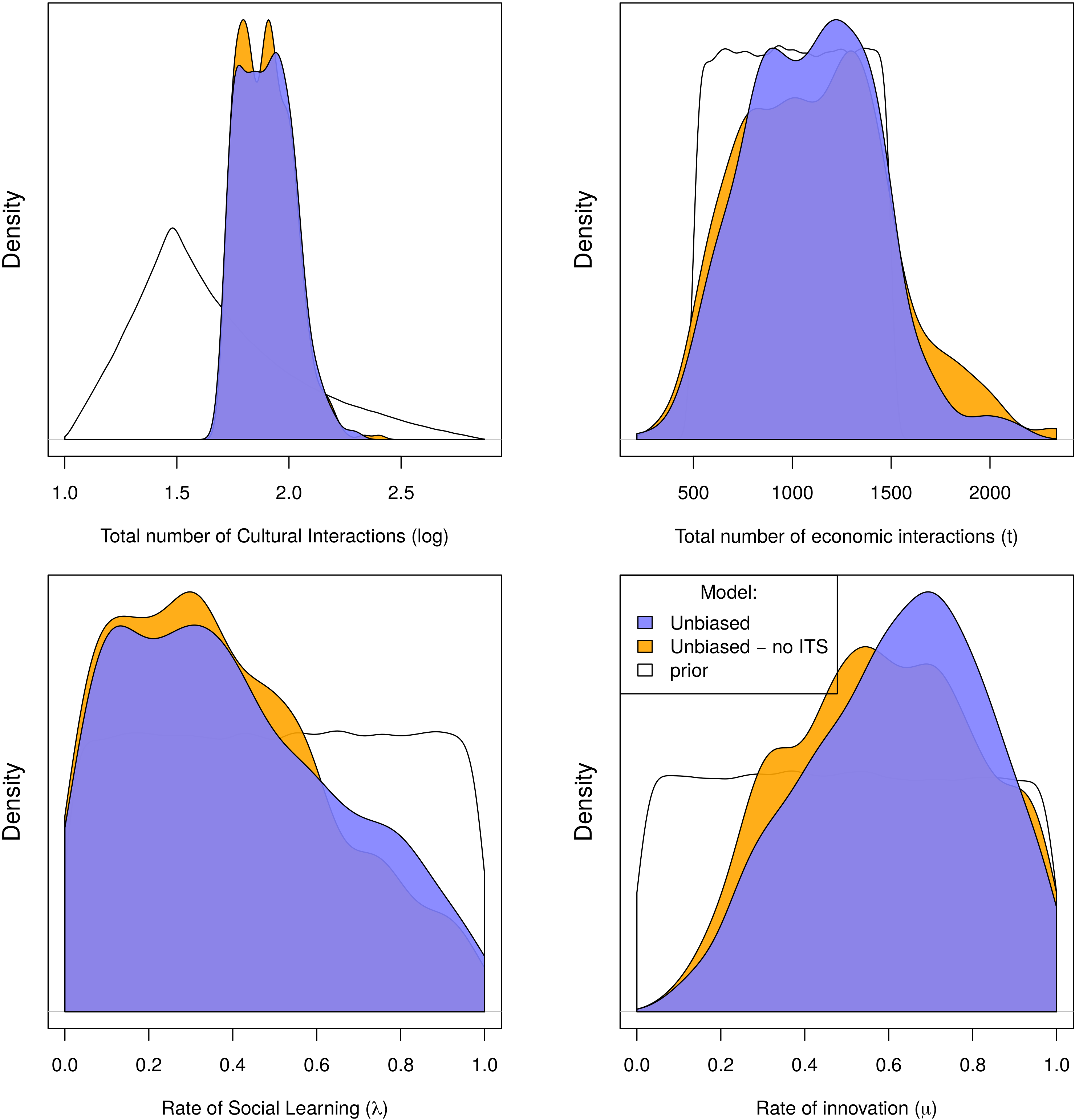

Supplement: S2 Fig — Comparison of the posterior for unbiased social learning with and without Italian Sigillata. The two experiments didn’t present any differences. (TIF) [file pone.0240414.s002.tif]
