# Supplementary material for: Tableware trade in the Roman East: Exploring cultural and economic transmission with agent-based modelling and approximate Bayesian computation
Source: PLoS One. 2020 Nov 25;15(11):e0240414. doi: 10.1371/journal.pone.0240414 (PMC7688115; doi:10.1371/journal.pone.0240414)
Supplement: S1 File — The full project, with the data, the code of all components used to run the simulation and analyses is registered: https://osf.io/s5mdw/. (GZ) [file pone.0240414.s003.gz › s5mdw-full/Archive of GitHub_ simoncarrignon-pandora.zip!/examples/gujarat/doc/Simulpast-CS1-ODD.pdf]

CS1: Hunter-Gatherer persistence in arid margins. The case of N Gujarat (India).

Table of Contents

1 Introduction.....2
2 Purpose.....2
3 Entities, state variables, scales.....2
3.1 Scales.....2
3.1.a Agent Scale.....2
3.1.b Time Scale.....3
3.1.c Space Scale.....3
3.2 Environment.....3
3.2.a Climate.....3
3.2.b Resources.....3
3.3 Agents.....5
3.3.a Hunter-Gatherer (HG).....5
3.3.b Agro-Pastoralists (AP) (work in progress).....6
4 Process overview and scheduling.....6
4.1 Precipitation calculation.....7
4.2 Biomass Yearly Production.....7
4.3 Daily processes.....7
4.3.a Biomass availability.....7
4.3.b Agent planning.....8
4.4 Adjustment of agent population size.....8
5 Design concepts.....9
5.1 Basic principles.....9
5.2 Emergence.....9
5.3 Adaptation.....9
5.4 Objectives.....9
5.5 Learning.....9
5.6 Prediction.....9
5.7 Sensing.....9
5.8 Interaction.....10
5.9 Stochasticity.....10
5.10 Collectives.....10
5.11 Observation.....10
6 Initialization.....10
6.1 Climate.....10
6.2 Environment.....10
6.3 Resources.....10
6.4 Agents.....10
7 Input data.....11
7.1 Rainfall.....11
7.2 Ground Model.....11
7.3 Hunter-Gatherer behavior.....11
8 Submodels.....13
8.1 Agent execution cycle.....13
9 References.....15

1 Introduction

Northern Gujarat is a marginal environment between the Thar Desert and the more fertile area of Saurashtra. This region is an ecotone, characterized by the seasonal influence of the monsoon where contrasting ecological niches are in tension and small climatic shifts can generate significant environmental changes, eventually affecting resource availability. Archaeological evidence points to the presence and possible coexistence in the area of groups of people with different resource management strategies and mobility behaviors: hunter-gatherers (HG); agropastoralists (AP).

The aim of this study is to model resource management and decision making among hunter-gatherer groups in this region to explore adaptive trajectories and performance in relation to a) environmental variability and b) the appearance of other specialized groups.

What factors play a role in HG persistence or disappearance in arid margins? Is the advent of agro-pastoral behaviour a big enough change to explain the disappearance of HG behaviour? Does climate variability affect HG behaviour?

The model description follows the ODD (Overview, Design concepts, Details) protocol for describing individual- and agent-based models (Grimm et al. 2006, 2010).

Note:
The modeling workflow is following these steps:

1. *Environmental settings and climate engine*
2. *Resources and energy flow*
3. *Socio-ecological behaviour of HG*
4. *Socio-ecological behaviour of AP*
5. *Cultural transmission*

This scaling approach includes three main theoretical and methodological research aspects:

1. *Human behavioral ecological approach and socio-ecological co-evolutionary framework*
2. *Model based vs rule based action planner of the agents*

Up to the date, steps 1, 2 and 3 have been developed . Work regarding AP models and cultural transmission is in progress.

2 Purpose

In our starting hypothesis HG groups are adapted to marked seasonality (represented by the monsoon) in the arid margins of northern Gujarat. We intend to explore HG resilience (Holling 1973, Carpenter et al. 2001) considering: a) the appearance of AP, b) climate variability.

3 Entities, state variables, scales

The model initially explores separately socio-ecological behaviours of two isolated populations: 1) Hunter-Gatherers (HG) agents and 2) Agro-Pastoralist (AP) agents. This separate simulation of the two groups is needed to obtain independent, coherent and consistent models of HG and AP decision making. Once the dynamics of the modeled systems are understood for each population, agents from the two populations will be combined in a single simulation execution. These will be considered as independent groups, and interaction between agents will be limited to other agents belonging to the same population.
Agents from one population will interact within a given territory. It is characterised in terms of: a) geographical information derived (height, slope), and b) landscape (soil types, resources). This territory and its characteristics will generate an environment that will allow to portray differences in strategies regarding settlement, mobility and resource use.

3.1 Scales

3.1.a Agent Scale

For both populations (AP and HG) the basic agent is defined as a couple (one woman and one man). This is considered to be the entity engaging in all decision-making processes and actions that will be modelled in the simulation.

3.1.b Time Scale

Time Scale for the simulation is one day. This time step is coherent with the granularity of agents' planning.

3.1.c Space Scale

The *spatial* resolution of the proposed simulation model is constrained by the resolution of available relevant geographic data and the nature of the agent mobility and resource gathering activities being modelled.

Hence, it was decided to use 31.5m x 31.5m cells, corresponding to ca. 1,000 square meters. First and most important, this is the level of resolution of the most detailed geographical information available for the area; second, this surface can fit the type of settlements recognised from the archaeological surveys.

3.2 Environment

The simulation environment is large enough to develop all potential processes defined by the model. It extends over an area of 25 Km x 25 Km (625 Km2). Space is represented as a regularly spaced grid of cells (a raster map). Each cell is a square of 31.5 m per side, and the total size of this environment translates into a space of 800 x 800 cells (25,200 m x 25,200 m).

The ground model includes elevation and land features. Elevation is determined by a Digital Elevation Model (DEM), a raster map containing the elevation value for each cell calculated from contemporary satellite imagery. Land characteristics are reduced to three elemental features:

- Water Body: represents rivers and lakes.
- Sand Dune: represents the top area of the dune, which can be settled. Home location of the agent will always be in a dune cell.
- Interdunal Soil: represents the interdune area where resources grow. The different land features do not seasonally change in extension but their productivity (in terms of moist content and therefore resources supported) does.

The cornerstone of our environmental modeling is the climatic engine. The climate module determines the quantity of rain that precipitates evenly on the landscape on every time step. Precipitation is used in conjunction with the terrain model to calculate the amount of biomass for each cell and season. The climate model is based on present-day, historical and palaeoclimatic data, as well as Holocene monsoon models.

3.2.a Climate

The focus on the co-evolution of resource utilization strategies within a particular environment requires to make explicit the potential variations in the landscape. In particular for our case study, the presence of the monsoon generates a strong seasonality (asymmetrical precipitation patterns).

Monsoon seasonality determines the presence of three critical "moments" in simulation time, each spanning 4 months. Therefore, the seasonal subdivision in three periods will be repeated in a cyclical way as follows:

- JJAS (rain season: high precipitation, high temperature, low evapotranspiration)
- ONDJ (post-Monsoon: low precipitation, cool temperature, medium evapotranspiration)
- FMAM (dry season: low precipitation, high temperature, high evapotranspiration)

It is important to note that any given "year" in the model starts with the beginning of the rain season (June). In fact, virtually all rain in the region is carried by the monsoon that falls between June and September (JJAS). Therefore, it is during the JJAS beginning that the totality of the generated yearly precipitation value will be calculated (following the Weibull distribution). No additional precipitation is considered for the remaining eight months of the year (ONDJ and FMAM).

3.2.b Resources

Each cell has a finite number of **resources** and a **type.** Any given cell can be:

Wild cell. Resource availability for each cell is calculated from the following variables:

- Yearly precipitation (rainfall, the Weibull distribution)
- Type of cell (Water Body, Interdune, Dune)
- Mean yearly Biomass per cell and type.
- Cell history (e.g. whether the cell was used for crop in a previous step or part of the resources were consumed before).

Wild resources include the total biomass that can be found in a cell (fauna and flora). Wild resources are exploited by HG agents engaging into foraging activities. Foraging includes activities such as hunting animals and gathering plants, fruits, seeds, etc. Indeed, from a literature review it is clear that secondary biomass production (animals) is directly related to primary biomass quantity. Moreover, as there is no interest in our simulation to explore gender-based labour division we decided to consider hunting and gathering as a single activity (foraging) without distinguishing between plant or animal utilization. In the light of this, it was decided to consider a value for wild cell (dune vs interdune) based on published information of primary biomass production in desert (dune) and savannah (interdune) biomes (after Kelly 1983 – Table 3).

CELL TYPE	Yearly Primary Biomass Production	Efficiency	Energy	KCal
Dune (desert)	700g/m²	13%	1820KJ/m²	435KCal
Interdune (savanna)	4000g/m²	23%	18400KJ/m²	4395KCal

Percentage of accessible resources calculated accordingly to Kelly (1983; Table 3, p. 284).

Cell area = 1000m²

1 g Primary Biomass = 20KJ

1 kcal = 4.184 KJ

Efficiency

The total primary biomass value does not constitute the entire primary biomass available for consumption to animals and humans. This value represents the entire biomass production including both edible (fruits, tubers some roots etc.) and non-edible (wood, stems, branches etc.) parts of the plants. The ratio of profitable biomass versus whole biomass will be the efficiency value specified in the above table that allow to calculate the energy effectively available for humans.

Domesticated cell (*work in progress)*

Domesticated plant resources define the density of domesticated plants found inside the cell. It is important to note that wild and domesticated types of resources are mutually exclusive: i.e. wild resources are not found on cultivated plots. These cells can have two different states:

- **Crop:** The cell is ready for agricultural uses (sow-harvest cycle).
- **Fallow:** The cell can't be used for agricultural uses, but is good for grazing.

The basic idea for a domestication cell is that of small scale shifting cultivation, in which a plot is cultivated following a cycle that includes abandonment so to allow soil properties to recover. A domesticated cell can be planted for a maximum of three consecutive years. It then needs to be abandoned for a minimum of three years (during which it will be considered as **Fallow**). After three years of abandonment the cell becomes wild and can be used again for agriculture. If a plot is abandoned before being cultivated for three consecutive years it will have to remain abandoned for the same amount of years it was worked. During these years it will remain fallow, after that it will become wild again.

Crop cells change temporary their state to **Fallow** during the **FMAM** season, where no agricultural uses can be executed. It turns to **Crop** again during **JJAS** season, and will be ready to sow at the end of it.

3.3 Agents

The following attributes have been chosen to account in our model for both HG and AP:

- **Children** – A collection of human entities. The number of children is bound by resource availability.
- **Home location** – The cell were the agent resides. It is the spatial centre of the activities carried out by the agent. A given cell can be shared by more than one agent from the same group, be it HG or AP. On the other hand, there is a physical limit (**Maximum Homes per cell**) in the number of agents that can settle the same cell (max 20 agents per cell, see HG Surrogates).
- **Relationships** – These are the connections between agents. The following types of relationship are considered:
    - **Acquaintance** – This is the result of having met at some time in the past. A numeric value is used to track the intensity of the relationship. This value changes with and depends on the actions of the involved agents.
    - **Next Of Kin** – Agents connected by family ties (the agents have some common ancestor). The closeness of the family tie is also modelled with an intensity value that remains constant throughout the agent´s life. The value is inversely proportional to the distance in the genealogical tree.
- **Surplus** – At any point in time, the amount of calories the agent did not use for survival.
- **Age** – A numeric variable that keeps track of how many time steps a given agent has been active in the simulation.
- **Food Needs** – A costant that sets the minimum calories a given agent needs in each time step. This survival threshold can be different for different types of agents.

3.3.a Hunter-Gatherer (HG)

The following attributes are specific for hunter-gatherers:

- **Home range** – The maximum distance an agent will travel in one day. This attribute restricts foraging activities by not permitting or making it anti-economic to engage in foraging in cells too far from the agent Home Location. Social activities are also limited in a similar way. The area enclosed in the circle of radius “Home range” is divided in a predefined number of sectors. Such division allows to model the idea of direction of exploration for the agent actions, while simplifying the decision-making process (an agent will choose to forage in one of the sectors, not particular cells). More details can be found below associated to the description of actions and the agent decision cycle submodel.

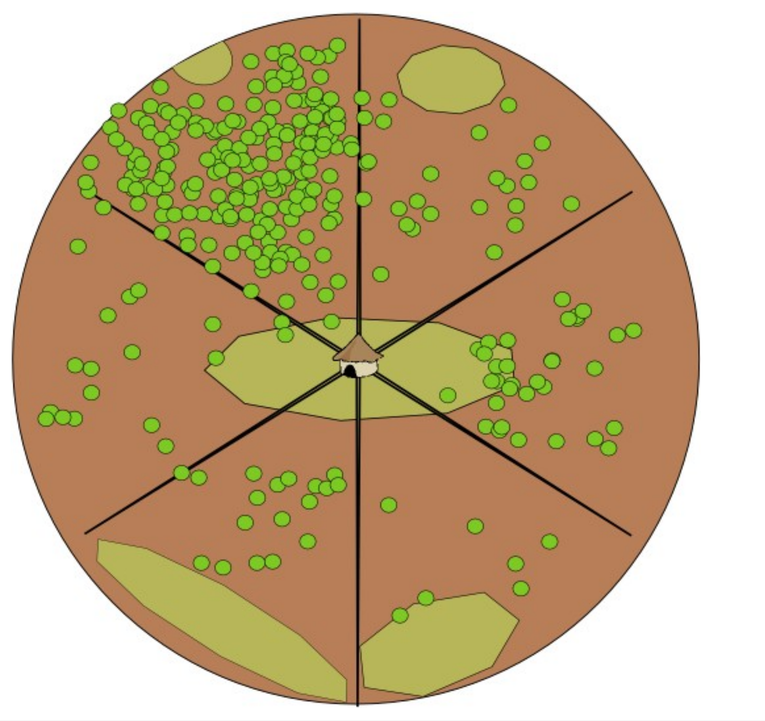

Home range division for foraging and moving home actions.

3.3.b Agro-Pastoralists (AP) *(work in progress)*

The following attributes are considered to be specific to agro-pastoralists:

- **Plot** – A cell cultivated by an agent.
- **Herd** – The quantity of domesticated animals that the agent owns. Although herds are resources for Agro-Pastoralists, they have been modelled as an attribute of the agent. The model keep track of the size of the agent's herd, and it is responsible for grazing the animals in order to maintain the number and get resources from the herd. This action will use wild and owned fallow cells inside the **Home Range**.
- **Maximum plot range** – The maximum distance in cells from **Home** that AP agents are allowed to go to set up an agricultural plot.
- **Maximum grazing range** – The maximum distance in cells from Home that AP agents are allowed to go for grazing.

AP agents may or may not have both plots and herds.

4 Process overview and scheduling

The execution has 2 scales. On the one hand, three processes ('Yearly Precipitation', 'biomass yearly production' and 'Population size adjustment') are executed once every year. On the other hand, agents decision-making processes use a daily time step. The simulation follows this schedule, beginning the first day of a JJAS season:

For each year:

1. Precipitation calculation
2. Biomass Yearly Production
3. For each day of the year:
    a. Daily biomass availability
    b. Agent planning:
        i. Knowledge update
        ii. Choice of actions
    c. Execution of agents actions
4. Population size adjustment

Details for each simulation phase are given hereafter.

4.1 Precipitation calculation

The total amount of rain is calculated as a random number following the Weibull distribution defined in section 8 (Input Data)

4.2 Biomass Yearly Production

The biomass that a cell will produce in an entire year is calculated from rainfall and mean year production for its particular type, provided by historical records.

We will consider a lineal relation between rain and biomass production for the sake of simplicity. The deviation of rain estimated for year from its mean will allow to interpolate the amount of biomass deviation from the yearly mean biomass. That is, if the mean of rain is 100 liters and the climate model produces 80 liters the deviation to apply is 20%, and for that year the biomass will be a 80% of the mean yearly production.

4.3 Daily processes

4.3.a Biomass availability

Yearly biomass production will not appear immediately in the cell in the first day of JJAS season. Resources will gradually increase under the accumulation of water until some days after the end of the season. It will slowly decrease in the following months, until the beginning of the next rainy season.

Next figure depicts this process as a triangle, being its Height (M) the peak of resources in a given cell, its Base (L, divided in L1=JJAS and L2=ONDJ+FMAM) the time span of one year, and its area (A) the yearly biomass production.

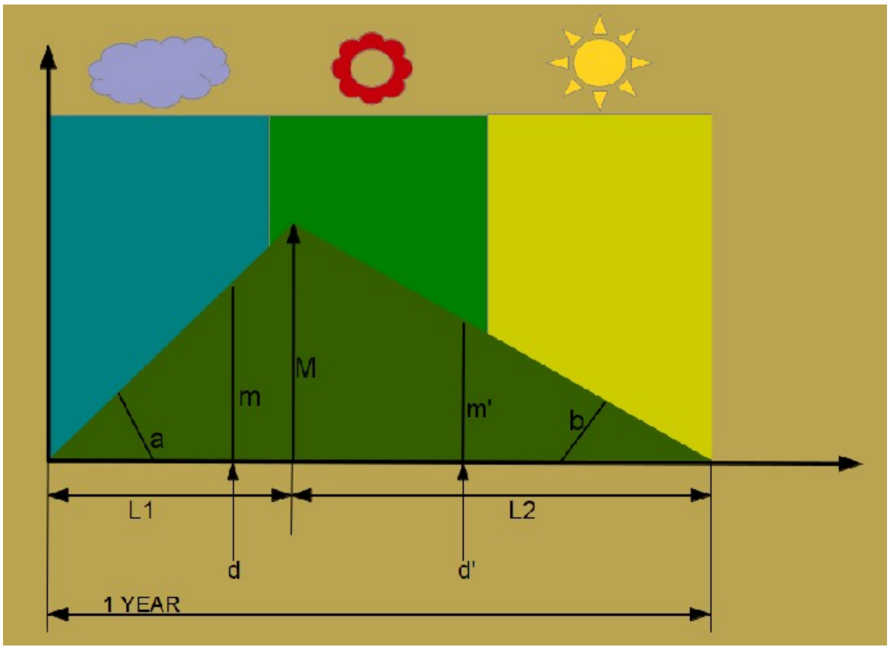

The biomass production follows this constraint:
Yearly Biomass Production = L1*M/2 + L2*M/2 = (L1+L2)M/2.

Peak M is calculated as:

$$A = B * H \div 2$$

$$A = (L1 * M \div 2) + (L2 * M \div 2)$$

$$A = (L1 + L2) * M \div 2$$

$$M = 2 * A \div (L1 + L2)$$

Finally we calculate the daily ratio of resource grow (JJAS) and decay (ONDJ-FMAM) for each cell as follows:

$$Grow = M \div L1$$
$$Decay = -(M \div L2)$$

4.3.b Agent planning

Each day the agent will update its knowledge about environment and choose a list of actions to execute (the decision-making process is defined in the Submodels section). The list of available actions is:

- **Common to HG & AP:**
  - **Move Home** - The agent moves from its current home location to set up a new one elsewhere inside Home Range. New home settlement will be chosen randomly between the dune cells from one of the sectors, the richest one. If needed, agricultural plots within the radius defined by the Maximum Plot Range attribute are also abandoned.
  - **Forage** – The agent does multiple walks of a bounded length fixed by the maximum activity per day. The walks are enclosed within its home range. From the visited cells, resource reward is retrieved based on biomass of the cells. The agent will halt the walk when reward achieves survival threshold. A walk comprises a daily foraging session.
  - **Ask for help** – The agent asks another agent, within its home range, for some calories. The probability of the other agent complying with the petition is proportional to the intensity of the relationship between the agents (much higher if the relationship is next of kin).
  - **Make gift** – The agent gives some calories to another agent (within their home range). This increases the intensity of the relationship between the agents.

- **AP particular actions:**
  - **Establish agricultural plot** – If the agent does not have an agricultural plot, it will stablish a new one. It must be an interdune cell where cannot be any previously existing plot established there.
  - **Abandon agricultural plot** – The agent abandons an already established agricultural plot. This action will be mandatory if the agricultural plot state has changed to Fallow due to being cultivated for three consecutive years.
  - **Sow** (during ONDJ season) – If the agent has a plot to cultivate, this field will be ready for sowing at the beginning of ONDJ (after the rain season).
  - **Harvest** (during ONDJ season) – By the end of the ONDJ season AP with a cultivated crop will reap and store the harvest. Resources available for the agent are accordingly updated, and excess production after the end of the year will be stored for consumption until the end of the following cool season
  - **Maintain plot** – The agent works on an established agricultural plot. There is a minimum of actions that need to be executed in order to get the optimal caloric yield from the plot. If this minimum is not reached, the yield will be slower.
  - **Maintain herd** - The agent feed and tend its animals. The number of executed actions of this type has two different outcomes: it regulates the size of the herd (i.e. lower values will seen this number decrease), and provides an income in terms of collected resources from the herd (milk, meat, etc.).

4.4 Adjustment of agent population size

This processes are executed for each agent:

1. **Age**. Agent ageing (increment human objects age).
2. **Death**. Every individual inside an agent will have a probability of dying (specially high during infancy) depending on current age.

3. **Removal**. If available resources are not enough to maintain the agent, it will be removed from simulation. This mechanism is meant to model both starvation and emigration events.
4. **Reproduction**. The probability of having new children depends on surplus availability for the agent, so for simplicity reproduction is not an agent's decision.
5. **Emancipation**. An agent with children coming of age will seek suitable matches amongst acquitances. Priority should be given to those acquaintances with higher surplus/better relationships.

5 Design concepts

5.1 Basic principles

The behavior defined in this model is derived from the Optimal Foraging Theory (OFT), developed within behavioral ecology. The main principle of OFT is the maximization of long-term energy gain. In other words, it is usually assumed that animals attempt to maximize the benefit to cost ratio. Evidence exists e.g. among great tits, birds that show relatively successful strategies in terms of OFT. Although it is doubtful whether humans attain the optimal rate of energy gain, they do succeed in improving their foraging efficiencies, or ”memorizing”.

5.2 Emergence

The most important emergent process that the system should see is the development of an equilibrium between HG and AP population sizes. This is the predicted outcome due to the fact that this equilibrium can be seen in archaeological and etnographical records.

5.3 Adaptation

Adaptive options for the agents are based on the complexity of the decision making process. Actions costs and outcomes are predefined following the model's configuration, but the order and the use of them is entirely an agent's choice. Following this reasoning, the different agents will try to adapt the dynamics of environment (both ecological and social) planning a different set of orders each time step.

A further step in this model will see mixed agents that are capable of choosing actions typical of HG and AP populations. Each agent will have different efficiency values for different actions, that will vary in time. In this way, the agent will also adapt specializing its actions.

5.4 Objectives

Following basic principles stated before, the objective for any agent is the survival of its individuals. This assumption is clearly from optimizing the system, as the different populations won't be guided by the mission of “colonizing” the entire landscape. Anyway, this outcome will be seen following evolutionary mechanisms and positive selection. Well adapted agents will have more possibilities to survive, thus creating more children and agents with similar cultural traits.

5.5 Learning

Learning processes will be modeled after cultural transmission concepts (i.e. vertical, oblique and horizontal mechanisms).

5.6 Prediction

Agents predict outcomes while evaluating and choosing possible actions. Every action has associated a cost and a possible outcome, that can be constant or stochastic (the latter designed to simulate risk). Depending on objectives, history of the agent and prediction the agent will decide the plan for the current time step.
Regarding these costs, two possible solutions have been discussed: (1) use calories, (2) use time spent. Option (2) sees agents as “batteries”, each time step or space step used by the agent (independently of the reason they need it for) the battery discharges a little; each time they forage, collect, harvest successfully the battery recharges (in terms of time and space) proportionally to the amount gained. HG can only store for few days, AP until the end of the following harvest (cool) season.

On the other hand, an agent does not keep track of previous rainfall values, so it is not able to predict the future state of the environment.

5.7 Sensing

An issue seldom addressed in the literature of ABM applications into Social Sciences is the fact that agents do not have perfect information on their environment.

With the agent architecture outlined above, it is quite simple to introduce the notion of a sensor model that mediates between the agent control procedure and the actual state of nearby cell objects. The introduction of this concept allows modeling in a nuanced and more realistic way agents' awareness of profitable cells, adding some complexity to the model. Agents posses an internal representation of their surroundings, causally connected but probably different from the actual surrounding conditions (i.e. the resource objects associated with cells). In addition, the agent´s model of the world can be complemented by the social structure of the model (sharing information between relatives and other acquaintances)

5.8 Interaction

The model is using a simple social interaction model, based on kinships and knowledge of near agents. It must be expanded using information from archaeology/anthropology in order to create social networks.

There exist also an indirect mechanism based on competition for land use. AgroPastoralists change the condition of particular cells from Wild to Domesticated/Fallow, decreasing the number of available cells for hunter/gathering. Finally, different types of agents can't share a given cell as Home.

5.9 Stochasticity

Stochasticity is used in three different concepts:

- Environment. Precipitation is calculated as a estochastical process following a Weibull distribution.
- Outcomes. Some actions have different outcomes depending on stochastic processes, like forage and harvest. It encapsulates the complex process of resources collection (i.e. risk, variability, etc.), and it is important due to the fact that Actions will be chosen depending on their outcomes and risk of failure.
- Life events. Death and reproduction are stochastic processes following realistic distributions.

5.10 Collectives

The agent, atom of the decision-making process, is itself a collective of different related individuals. Future work in the model will introduce different emergent collectives following social interaction.

5.11 Observation

Population dynamics and land uses are the most important concepts to derive from the model.

6 Initialization

Initial state of the model is divided by entities:

6.1 Climate

Rainfall yearly precipitation is an stochastically value calculated from input data, as seen in section 8. Calculated values depend on the initialization seed used in the random number generation, that is stored as a parameter of the model's configuration.

6.2 Environment

Ground Model and land features are raster maps created from real data (see also section 8). The model is able to load any raster map with correct values. This process is done during initialization time from the file specified in the configuration.

6.3 Resources

The conversion functions that create available biomass from landscape and rainfall for each cell (both Wild and Domesticated) use parameters specified in the configuration. They are based on published research; nevertheless they can be modified in order to explore different worlds.

6.4 Agents

Several parameters can be changed from the configuration. These values are loaded during init time, and remain stable during the entire execution:

- Life-event related:
  - Reproduction Probability (HG/AP): Constant
  - Dying age (HG/AP): Stochastic distribution
  - Adulthood (HG/AP): Constant
  - Maximum Homes per cell (HG/AP): Number of agents that share cell as Home.
- Resource related:
  - Home Range (HG): Constant
  - Maximum Plot Range (AP): Constant
  - Maximum Grazing Range (AP): Constant

This first model will use arbitrarily chosen values for all these parameters, but they will be adjusted (and justified) according to present research.

7 Input data

7.1 Rainfall

This model uses as a main environmental engine rainfall yearly precipitation. Data for precipitation rate is extracted from historical data at IITM covering a total of 138 years (1844 to 1982). The climate engine is defined as a probability distribution, from which the total amount of rain fallen during a year is derived. We have defined a Weibull distribution with parameters shape=2.63, scale=5278.81, to be the best fit for the rainfall dataset we have acquired.

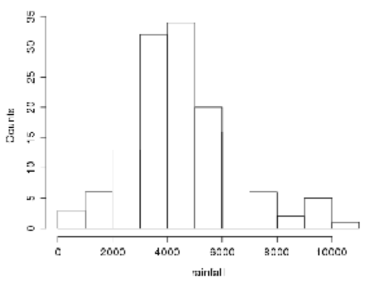	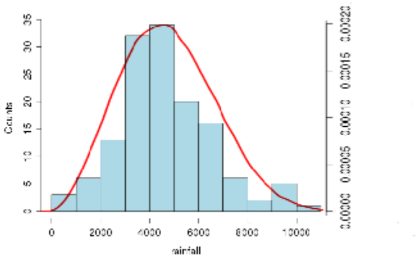
Histogram from precipitation samples in the considered years.	Weibull Distribution, calculated from previous precipitation histogram (shape=2.63, scale=5278.81).

7.2 Ground Model

This model is derived from ASTER satellite imagery (combining pre- and post-monsoon imagery) and includes DEM and land features. ASTER data are elaborated using unsupervised classification and clustered in the 3 classes (water body, interdunal and dunal area). The model is exported as a Raster map.

7.3 Hunter-Gatherer behavior

Archaeological data are always incomplete in their nature and from the analysis of data archaeologists can only infer few behavioural patterns. Therefore, the use of ethnoarchaeology is useful to be able to infer HG behaviours from the observation of historical or modern-day populations in similar ecological setting. IN the case of our study, we rely on anthropological studies of HG societies inhabiting an environment similar (specifically in terms of productivity and agents' energetic expenditure) to the one we reconstructed for Gujarat. In doing so we assume that similar environmental conditions and similar technologies (hunting tools, building strategies etc) would lead to similar socio-ecological behaviours. Although there are a few HG groups that inhabits an area geographically close to North Gujarat (the Van Vargis, see Nagar 2008), these communities have a high degree of interaction with and dependency to settled agricultural communities for their subsistence strategies. In our vision this

constitutes a strong bias towards the use of this society as parallel to the modelling of our HG agent. Therefore we decide to use as surrogates of our HG African groups of the San communities.

The San (especially the G/wi and G//ana groups of Botswana) represent the best-fitting parallel in terms of ecosystem inhabited and exploited (Tanaka and Sugawara 1996). These groups are found on a flat plateau in the central part of the Kalahari desert. The landscape morphology is characterised by fossil rivers and traces of sand dunes. Rainfall is concentrated in the summer months with a bout 400 mm annual average. The vegetation of the area is dominated by plants of the Gramineae family (grasses) and a mixture of shrubs of the same genus/families that are found in North Gujarat.

**HUNTER GATHERER's SURROGATE**			
**Surrogate Group**	**Description**	**Item**	**Info**
San<br>(G/wi, G//ana)	*From Botswana (central Kalahari Desert, Africa)*	Lifestyle	Hunter-gatherers
		Band	[25..85] members.<br>*Formation and fission dependent on drought, disease, overpopulation and resource overloading.*
	Some households have quasi extensions of one or even two elderly parents of one or both spouses. Although separate shelters are occupied by the two senior generations, food, firewood, and other commodities are shared to some extent and the whole unit migrates together between campsites, into winter isolation, and between bands.	Family	Husband + 1 or more wifes + under puberty children (older sons stay in the bachelor's hut).
		Network	Kinship and affinity.
		Settlement	[1..20] families, mean=10
	Guided by the interband intelligence network, the founder leads a party of men on an extended hunting trip into country offering the most likely prospect of sufficient resources. Later they take their families with them and spend a month or more away from the parent band, returning before the annual breakup in winter. The following summer, once the wet season is established, the pioneers make their first visit and continue to	Movement	Dependent of the band.<br>A movement group is formed based on kinship and friendship.<br>Done at the end of summer.

	spend longer and longer periods in the new territory. The composition of the group changes to some extent as some withdraw and elect to remain with the parent band or to move to other established bands, and their places are taken by others. As the absences of the pioneers in their territory grow longer and more frequent, the separate identity of their group emerges until, eventually, it is recognized as an autonomous band.		
		Hunting	* 2 strategies: day sortie, biltong.<br>* Pair of hunters.<br>* Stick to an area fixed the previous night with other pairs.<br>* Everybody helps (information) the hunters track the games.<br>* <5 pairs go hunting the same day.<br>* Hunt range around the camp = [700..800]km².<br>* Could spend night far from home (>20km away otherwise will return).
		Gathering	80% of total reward.<br>[1h walking (if good tsama season)...6h walking]<br>[3.5kg..5kg] per day and individual.
		Working day	* 4h39' / working day.<br>* 9h away from camp = 4.5 h working + 4.5 leisure<br>* [10:30 .. 16:00] = rest

8 Submodels

8.1 Agent execution cycle

The majority of Agent-Based Models mix knowledge acquisition, decision-making and execution in the same phase of an agent's execution. This choice is useful if we deal with agents with simple decision-making processes where the choice of behaviors is predefined beforehand. However, this classical

approach to ABM has a major drawback, and is the fact that the agent will have scripted strategies, and for this reason it won't be able to choose strategies different from the ones defined there.
The model proposed here uses goal-oriented agents. During each time step every agent will update its knowledge about the environment (possibly including other agents). This action is combined with a set of goals and available actions, in order to let the agent choose which plan of actions wants to execute. Several factors can be used to enrich the process:

- Agent's goals and agent's preferences referred to the choice of particular actions
- The information that the agent perceives from the environment and the reliability of such information.
- The information collected from other Agents, as well as its reliability.
- The feedback the agent receives from engaging into a given activity.

In this particular model the goal of every agent is to maintain alive its individuals, and the potential actions are the ones defined in the document for each type (Hunter-Gatherer and Agro-Pastoralist). Each of these actions have a cost (in time) and a potential reward in relation with agent's objectives. This will be useful to order possible actions from less to more valuable. Finally, the plan of each agent is executed, thus evaluating the outcomes of the different actions that every one of them has chosen.
The execution of the agents during each time step is divided in three different phases:

1. **Knowledge update**

The agent collects information from the environment, and creates an individual representation of the world using its preferences and objectives. Hunter-Gatherer agents will calculate the amount of biomass available in each directional sector, as well as potential settlement zones.

2. **Creation of the plan**

The agent will decide which actions it will execute once knowledge has been collected. The entire collection of plans is organized as a tree of potential actions, and the agent evaluates the best possible course of actions given the current state of the world and its own goals.

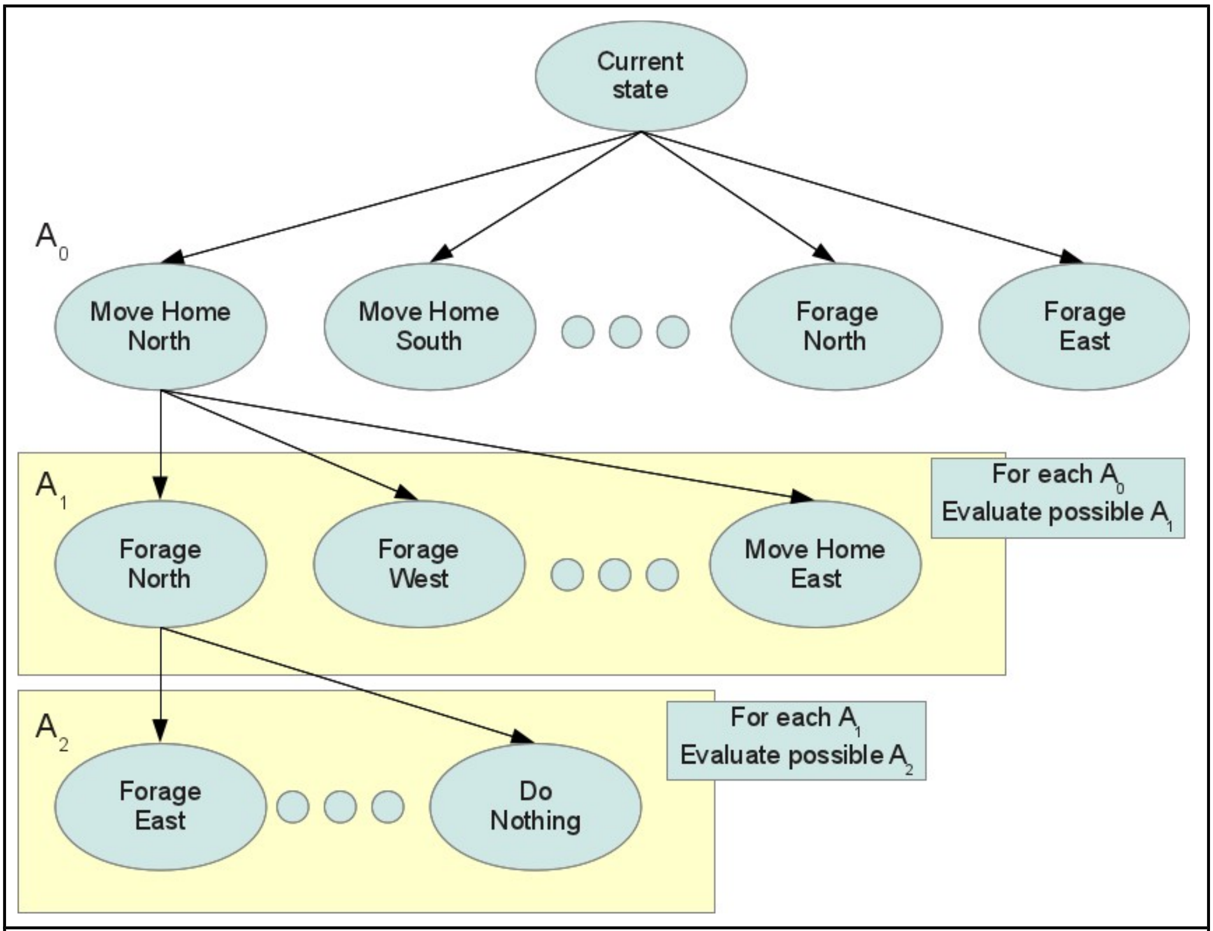

Tree of possible actions for a particular Hunter-Gatherer agent.

3. **Actions execution**

Once every agent has decided its own plan, all of them are executed sequentially following a randomized order.

9 References

- Carpenter, Steve, Brian Walker, J. Marty Anderies, and Nick Abel (2001). “From Metaphor to Measurement: Resilience of What to What?” Ecosystems 4: 765–781
- Grimm V, Berger U, Bastiansen F, Eliassen S, Ginot V, Giske J, Goss-Custard J, Grand T, Heinz S K, Huse G, Huth A, Jepsen J U, Jørgensen C, Mooij W M, Müller B, Pe’er G, Piou C, Railsback S F, Robbins A M, Robbins M M, Rossmanith E, Rüger N, Strand E, Souissi S, Stillman R A, Vabø R, Visser U and DeAngelis D L (2006). “A standard protocol for describing individual-based and agent-based models”. Ecological Modelling 198 (1-2), 115-126.
- Grimm V, Berger U, DeAngelis D L, Polhill J G, Giske J and Railsback S F (2010). “The ODD protocol: A review and first update”. Ecological Modelling 221 (23), 2760-2768.
- Holling CS (1973). “Resilience and Stability of Ecological Systems Annual Review of Ecology and Systematics Vol. 4: 1-23.
- Kelly, R.L. (1983). Huter-gatherer mobility strategies. Journal of Anthropological Research, 39(3), 277-306.
- Nagar, M. 2008. Hunter-gatherers of North and Central India: an Ethnoarchaeological Study. BAR International Series 1749. Arcaheopress: Oxford.
- Tanaka, J., Sugawara, K. (1996). The /Gui and //Gana of Botswana. In In: Lee, R.B. And Daly, R. (eds). The Cambridge Encyclopedia of Hunters and Gatherers, CUP, Cambridge, pp. 195-199.
