# Supplementary material for: Tableware trade in the Roman East: Exploring cultural and economic transmission with agent-based modelling and approximate Bayesian computation
Source: PLoS One. 2020 Nov 25;15(11):e0240414. doi: 10.1371/journal.pone.0240414 (PMC7688115; doi:10.1371/journal.pone.0240414)
Supplement: S1 File — The full project, with the data, the code of all components used to run the simulation and analyses is registered: https://osf.io/s5mdw/. (GZ) [file pone.0240414.s003.gz › s5mdw-full/Archive of GitHub_ simoncarrignon-pandora.zip!/examples/gujarat/libmdp/ctp/results-opt.pdf]

CTP: April 29, 2010 10:56 (based on 1000 runs)

Cost

**solver**	ours 10 #00	ours 10 #01	ours 10 #02	ours 10 #03	ours 10 #04	ours 10 #05
DIJ	101.364±3.723	115.973±3.230	124.395±3.284	53.811±2.820	85.946±2.320	102.468±3.498
HOP:N:10000:N:Y:Y:N	101.982±3.811	99.958±2.281	114.796±3.000	55.831±2.770	86.921±2.257	95.720±2.608
MC:N:10000:N:Y:Y:N	101.793±3.791	100.251±2.265	115.897±3.108	53.495±2.680	86.167±2.311	91.571±2.916
UCT:N:10000:0.1:Y:Y:3:20:Y	101.351±3.723	99.929±2.270	115.238±3.043	53.445±2.690	86.132±2.332	92.609±2.851
UCT:N:10000:1:Y:N:0	114.881±3.866	102.568±2.359	127.220±3.355	58.038±2.810	89.325±2.474	111.525±3.549
ldfs	$_{1}$ 122.173±nan	$_{1}$ 120.509±nan	$_{1}$ 123.626±nan	$_{1}$ 55.225±nan	$_{1}$ 91.981±nan	$_{1}$ 99.845±nan

**solver**	ours 10 #06	ours 10 #07	ours 10 #08	ours 10 #09	ours 11 #00	ours 11 #01
DIJ	76.250±2.649	72.814±1.490	68.142±2.985	76.914±2.788	149.277±3.424	140.153±4.106
HOP:N:10000:N:Y:Y:N	66.792±1.778	72.756±1.488	66.492±2.675	75.228±2.664	108.297±1.489	115.528±2.572
MC:N:10000:N:Y:Y:N	66.792±1.778	72.765±1.489	66.452±2.662	75.289±2.672	109.232±1.630	125.205±2.577
UCT:N:10000:0.1:Y:Y:3:20:Y	66.701±1.769	74.673±1.573	66.553±2.685	75.403±2.681	108.884±1.569	113.262±2.561
UCT:N:10000:1:Y:N:0	83.328±2.548	87.080±1.938	72.084±3.463	76.734±2.750	131.561±3.017	127.328±3.377
ldfs	$_{1}$ 72.946±nan	$_{1}$ 79.934±nan	$_{1}$ 79.834±nan	$_{1}$ 91.215±nan	$_{1}$ 115.556±nan	$_{1}$ 132.485±nan

**solver**	ours 11 #02	ours 11 #03	ours 11 #04	ours 11 #05	ours 11 #06	ours 11 #07
DIJ	109.290±3.432	95.123±3.022	90.253±2.443	95.894±4.054	73.066±3.649	93.669±2.805
HOP:N:10000:N:Y:Y:N	102.529±2.843	91.862±2.911	83.160±2.164	75.256±2.863	62.672±2.527	91.952±2.554
MC:N:10000:N:Y:Y:N	102.625±2.855	88.746±2.631	83.520±2.180	80.930±2.910	62.944±2.576	91.048±2.531
UCT:N:10000:0.1:Y:Y:3:20:Y	102.222±3.001	88.760±2.616	87.923±2.123	75.065±2.869	62.919±2.545	91.108±2.517
UCT:N:10000:1:Y:N:0	107.956±3.057	97.702±2.904	103.034±2.769	78.722±3.099	80.737±3.767	93.551±2.740
ldfs	$_{1}$ 103.023±nan	$_{1}$ 95.884±nan	$_{1}$ 91.463±nan	$_{1}$ 102.458±nan	$_{1}$ 75.478±nan	$_{1}$ 103.147±nan

**solver**	ours 11 #08	ours 11 #09	ours 5 #00	ours 5 #01	ours 5 #02	ours 5 #03
DIJ	75.897±3.294	86.650±3.270	80.996±3.429	26.847±1.086	64.617±1.429	44.169±2.232
HOP:N:10000:N:Y:Y:N	72.427±2.647	86.864±3.284	76.761±2.810	27.722±1.241	53.855±0.633	40.873±1.123
MC:N:10000:N:Y:Y:N	72.259±2.620	85.952±3.229	76.761±2.810	27.722±1.241	53.855±0.633	41.197±0.573
UCT:N:10000:0.1:Y:Y:3:20:Y	72.084±2.580	86.640±3.279	76.761±2.810	26.815±1.083	53.855±0.633	41.034±0.634
UCT:N:10000:1:Y:N:0	74.263±3.079	90.942±3.459	76.761±2.810	26.815±1.083	53.830±0.620	40.929±0.697
ldfs	$_{1}$ 91.513±nan	$_{1}$ 112.662±nan	$_{1}$ 79.587±nan	$_{1}$ 30.542±nan	$_{1}$ 56.500±nan	$_{1}$ 42.255±nan

**solver**	ours 5 #04	ours 5 #05	ours 5 #06	ours 5 #07	ours 5 #08	ours 5 #09
DIJ	40.377±1.583	35.287±1.000	44.658±1.236	44.677±1.283	62.594±3.748	48.022±3.051
HOP:N:10000:N:Y:Y:N	41.050±1.547	31.795±0.815	44.703±1.241	44.677±1.283	52.657±2.831	46.888±2.993
MC:N:10000:N:Y:Y:N	40.478±1.559	31.795±0.815	44.733±1.244	44.677±1.283	52.657±2.831	46.888±2.993
UCT:N:10000:0.1:Y:Y:3:20:Y	40.471±1.558	31.807±0.816	44.594±1.216	44.677±1.283	52.657±2.831	46.888±2.993
UCT:N:10000:1:Y:N:0	40.919±1.572	31.795±0.815	44.658±1.236	44.677±1.283	52.657±2.831	46.888±2.993
ldfs	$_{1}$ 40.835±nan	$_{1}$ 38.435±nan	$_{1}$ 50.188±nan	$_{1}$ 46.295±nan	$_{1}$ 104.301±nan	$_{1}$ 80.370±nan

**solver**	ours 6 #00	ours 6 #01	ours 6 #02	ours 6 #03	ours 6 #04	ours 6 #05
DIJ	94.896±3.248	48.062±2.225	29.514±0.641	56.917±2.227	79.577±1.544	78.926±1.714
HOP:N:10000:N:Y:Y:N	94.477±3.218	47.040±1.942	29.579±0.671	57.212±1.981	80.861±1.158	76.495±1.037
MC:N:10000:N:Y:Y:N	94.477±3.218	46.326±1.884	29.606±0.686	55.937±1.919	80.861±1.158	79.420±1.441
UCT:N:10000:0.1:Y:Y:3:20:Y	94.796±3.251	45.258±2.013	29.613±0.680	55.977±1.937	79.343±1.382	76.252±1.388
UCT:N:10000:1:Y:N:0	94.504±3.219	45.280±2.013	29.477±0.619	56.098±2.018	78.342±1.464	75.957±1.320
ldfs	$_{1}$ 107.570±nan	$_{1}$ 49.998±nan	$_{1}$ 31.356±nan	$_{1}$ 62.059±nan	$_{1}$ 81.275±nan	$_{1}$ 72.668±nan

**solver**	ours 6 #06	ours 6 #07	ours 6 #08	ours 6 #09	ours 7 #00	ours 7 #01
DIJ	25.682±1.844	52.493±0.535	56.049±1.521	42.907±2.451	98.778±3.904	35.184±0.953
HOP:N:10000:N:Y:Y:N	25.420±1.828	50.843±0.256	56.049±1.521	39.513±1.940	100.943±2.819	34.984±0.888
MC:N:10000:N:Y:Y:N	25.140±1.787	50.843±0.256	56.049±1.521	39.513±1.940	101.896±3.337	34.984±0.888
UCT:N:10000:0.1:Y:Y:3:20:Y	25.095±1.780	50.843±0.256	56.049±1.521	39.513±1.940	98.287±3.765	34.984±0.888
UCT:N:10000:1:Y:N:0	25.126±1.787	50.841±0.256	56.049±1.521	39.513±1.940	98.874±3.810	34.984±0.888
ldfs	$_{1}$ 29.969±nan	$_{1}$ 51.180±nan	$_{1}$ 65.313±nan	$_{1}$ 53.374±nan	$_{1}$ 106.552±nan	$_{1}$ 38.471±nan

**solver**	ours 7 #02	ours 7 #03	ours 7 #04	ours 7 #05	ours 7 #06	ours 7 #07
DIJ	89.251±3.566	56.732±2.024	78.705±2.083	100.641±2.161	70.212±1.137	80.453±2.044
HOP:N:10000:N:Y:Y:N	86.337±2.731	54.693±1.707	78.788±2.080	107.577±2.233	67.541±0.907	80.361±2.020
MC:N:10000:N:Y:Y:N	86.227±2.768	55.578±1.765	80.973±2.106	$_{999}$ 107.717±2.225	67.340±0.946	81.305±2.004
UCT:N:10000:0.1:Y:Y:3:20:Y	84.095±3.188	56.278±1.746	79.033±2.074	100.361±2.115	67.340±0.946	80.259±2.025
UCT:N:10000:1:Y:N:0	86.218±3.126	55.989±1.739	80.654±2.098	101.526±2.117	68.219±0.992	82.584±1.960
ldfs	$_{1}$ 92.807±nan	$_{1}$ 53.830±nan	$_{1}$ 80.435±nan	$_{1}$ 127.100±nan	$_{1}$ 72.180±nan	$_{1}$ 83.830±nan

**solver**	ours 7 #08	ours 7 #09	ours 8 #00	ours 8 #01	ours 8 #02	ours 8 #03
DIJ	35.607±2.278	46.405±1.259	121.034±3.696	72.380±2.610	94.187±2.366	66.898±2.707
HOP:N:10000:N:Y:Y:N	34.408±2.122	46.408±1.260	120.355±2.996	53.415±0.980	96.276±1.650	62.098±2.553
MC:N:10000:N:Y:Y:N	34.408±2.122	46.408±1.260	112.426±3.691	56.077±0.906	91.228±2.101	62.877±2.509
UCT:N:10000:0.1:Y:Y:3:20:Y	34.408±2.122	46.478±1.255	112.460±3.691	53.982±1.023	91.759±2.184	61.550±2.434
UCT:N:10000:1:Y:N:0	34.408±2.122	49.044±1.091	113.382±3.798	55.430±0.959	96.000±2.077	74.391±2.661
ldfs	$_{1}$ 37.315±nan	$_{1}$ 60.603±nan	$_{1}$ 124.343±nan	$_{1}$ 56.957±nan	$_{1}$ 95.226±nan	$_{1}$ 70.995±nan

**solver**	ours 8 #04	ours 8 #05	ours 8 #06	ours 8 #07	ours 8 #08	ours 8 #09
DIJ	76.555±2.039	38.202±1.934	68.156±3.535	83.569±2.878	45.899±2.259	51.070±1.468
HOP:N:10000:N:Y:Y:N	75.472±1.924	37.579±1.830	69.311±3.633	76.404±1.703	45.768±2.254	48.016±1.002
MC:N:10000:N:Y:Y:N	75.921±1.928	37.551±1.827	66.969±3.395	72.895±1.934	45.768±2.254	47.986±0.984
UCT:N:10000:0.1:Y:Y:3:20:Y	76.270±1.918	37.807±1.866	67.195±3.437	72.980±1.867	45.678±2.231	48.001±0.993
UCT:N:10000:1:Y:N:0	80.425±1.985	38.884±1.942	67.309±3.412	72.547±1.762	47.850±2.257	48.774±1.047
ldfs	$_{1}$ 84.111±nan	$_{1}$ 50.758±nan	$_{1}$ 81.171±nan	$_{1}$ 93.398±nan	$_{1}$ 65.467±nan	$_{1}$ 59.251±nan

**solver**	ours 9 #00	ours 9 #01	ours 9 #02	ours 9 #03	ours 9 #04	ours 9 #05
DIJ	110.091±2.577	57.964±2.962	103.962±3.050	54.569±1.543	64.392±2.327	117.941±3.583
HOP:N:10000:N:Y:Y:N	92.588±2.057	54.821±2.475	104.020±2.792	53.395±1.510	66.132±2.527	102.194±2.198
MC:N:10000:N:Y:Y:N	92.931±2.100	56.834±2.721	101.761±2.636	53.393±1.516	64.590±2.438	111.995±2.627
UCT:N:10000:0.1:Y:Y:3:20:Y	93.008±2.101	54.514±2.472	101.310±2.710	53.505±1.508	63.886±2.317	108.315±2.356
UCT:N:10000:1:Y:N:0	96.431±2.331	59.702±2.607	103.263±2.687	59.795±1.370	64.959±2.429	107.628±2.411
ldfs	$_{1}$ 110.889±nan	$_{1}$ 61.843±nan	$_{1}$ 114.987±nan	$_{1}$ 53.857±nan	$_{1}$ 76.579±nan	$_{1}$ 121.039±nan

**solver**	ours 9 #06	ours 9 #07	ours 9 #08	ours 9 #09
DIJ	108.258±4.347	38.295±1.669	63.909±3.500	46.992±1.498
HOP:N:10000:N:Y:Y:N	52.681±1.552	38.186±1.675	62.024±3.140	47.007±1.501
MC:N:10000:N:Y:Y:N	53.264±1.730	$_{999}$ 38.213±1.679	61.773±3.112	47.007±1.501
UCT:N:10000:0.1:Y:Y:3:20:Y	52.634±1.531	38.100±1.667	61.746±3.185	46.992±1.498
UCT:N:10000:1:Y:N:0	55.385±1.985	39.036±1.766	66.221±3.653	47.754±1.565
ldfs	$_{1}$ 64.975±nan	$_{1}$ 48.297±nan	$_{1}$ 87.405±nan	$_{1}$ 69.739±nan
